# Gut Hi-C metagenomes of severe COVID-19 patients: bacteria and yeast involved in gut-lung axis

**DOI:** 10.1128/msphere.00139-26

**Published:** 2026-05-19

**Authors:** Anastasia Z. Revel-Muroz, Ignat V. Sonets, Alexander S. Chistyakov, Petr A. Vasiluev, Yury A. Surovoy, Valeriia A. Ivanova, Liubov I. Kozlovskaya, Olga E. Khokhlova, Mikhail V. Fursov, Nadezhda K. Fursova, Sergey V. Ulianov, Alexander V. Tyakht

**Affiliations:** 1 Institute of Gene Biology Russian Academy of Sciences, Moscow, Russia; 2 Research Center for Medical Genetics, Moscow, Russia; 3 I.I. Mechnikov Research Institute for Vaccines and Sera, Moscow, Russia; 4 Faculty of Medicine, M.V. Lomonosov Moscow State University, Moscow, Russia; 5 Clinical Hospital #1 MEDSI, Otradnoe, Moscow Region, Russia; 6 FSASI Chumakov FSC R&D IBP RAS (Institute of Poliomyelitis), Moscow, Russia; 7 Sechenov First Moscow State Medical University, Moscow, Russia; 8 State Research Center for Applied Microbiology and Biotechnology, Obolensk, Russia; 9 Department of Molecular Biology, Faculty of Biology, M.V. Lomonosov Moscow State University, Moscow, Russia; Universita degli Studi di Napoli Federico II, Naples, Italy

**Keywords:** Hi-C metagenomics, gut microbiome, ICU, gut-lung axis, *Klebsiella*, *Candida*, plasmid transfer, drug resistance, COVID-19

## Abstract

Antimicrobial resistance (AMR) poses a critical threat to global health, particularly in intensive care units, where vulnerable patients are frequently exposed to multidrug-resistant microorganisms. The human gut microbiome serves as a key reservoir for AMR genes, which can disseminate to other body sites, including the lungs, especially during severe illness. We applied Hi-C metagenomics to stool samples from 11 critically ill COVID-19 patients and analyzed microbial isolates from their lungs to investigate intra-host transmission of AMR genes. Plasmid-resolved microbial interaction networks revealed AMR gene sharing across 13 bacterial genera, primarily from Firmicutes and Proteobacteria, with evidence of plasmid-mediated transfer across phylum boundaries and between gut and lung compartments. Notably, we identified genetically identical *Klebsiella pneumoniae* strains colonizing both the gut and lungs of a single patient, as well as shared plasmids carrying *qnrS-1* and *blaCTX-M-231* resistance genes between gut *Escherichia coli* and lung *K. pneumoniae*. In addition to bacterial pathogens, *Candida* yeast species isolated from both niches harbored resistance genes to multiple antifungal classes, including azoles. These findings underscore the dynamic, cross-compartmental nature of AMR dissemination within the human body and highlight the importance of integrative surveillance strategies to control resistance in clinical settings.

**IMPORTANCE:**

While COVID-19 itself caused severe illness, many deaths were ultimately due to secondary microbial infections—often worsened by antibiotic resistance. Plasmids, which shuttle resistance genes between bacterial species, are key players in their spread, yet their roles in transmission, especially across body sites such as the gut and lungs, are to be elucidated. The use of Hi-C metagenomics allowed us to map bacterium-plasmid links in the guts of severe COVID-19 patients and reconstruct high-quality genomes of opportunistic fungi. Comparing these with lung-derived isolate genomes, we gained insight into possible intra-host dissemination routes of resistance genes. Preparing for future pandemics will require not only rapid pathogen detection but also tools to monitor microbiome health and resistance dynamics, and understanding how treatments and microbial imbalances shape infection risks.

## INTRODUCTION

COVID-19, a viral infection that emerged in 2019, has had a profound global impact, affecting millions and frequently leading to severe complications. Bacterial infections are common comorbidities in COVID-19 cases—especially in severe and critically ill patients—and significantly increase mortality risk (1–3). Although the COVID-19 pandemic has subsided and the virus has become less virulent, the potential for future pandemics involving secondary bacterial infections remains. Therefore, investigating these bacterial infections is of continued importance.

The gut microbiome has been identified as a significant determinant of hospitalization duration and clinical outcomes in critically ill patients. Dysbiosis of the gut microbiota contributes to an increased risk of bacterial co-infections, which can worsen disease severity and delay recovery (4–7). In intensive care units (ICUs), prolonged antibiotic use drastically alters microbial communities, decreasing diversity and promoting the proliferation of opportunistic taxa (8–10). Notably, severe COVID-19 cases have also been associated with similar microbiome alterations (3–5). This microbial imbalance heightens the risk of hospital-acquired infections. Many of these opportunistic pathogens harbor resistance genes against modern antibiotics. Particularly concerning are gram-negative strains of *Escherichia coli* and *Klebsiella pneumoniae* resistant to broad-spectrum beta-lactams and carbapenems, as well as vancomycin-resistant *Enterococcus faecium* and methicillin-resistant *Staphylococcus aureus* (11, 12). The development of multidrug-resistant (MDR) secondary infections substantially increases the mortality of ICU patients with different diagnoses (12, 13).

Modulating the gut microbiome may represent a promising adjunctive strategy for improving outcomes in COVID-19 and other severe respiratory infections through beneficial microbial metabolites, diet, probiotics, and emerging approaches such as fecal microbiota transplantation (14, 15). Clinical and review data suggest that plant-rich diets, probiotics, and other microbiome-directed interventions can alleviate symptoms, support recovery, and restore microbial balance, although evidence remains limited and heterogeneous (14–18). At the same time, antibiotic use in severe respiratory infections must be carefully balanced, as broad-spectrum regimens can deplete protective taxa and promote antimicrobial resistance (AMR) (19), whereas targeted approaches may better preserve gut-lung homeostasis (20).

Antimicrobial resistance in these pathogens is often linked to plasmids carrying resistance genes (21). A key feature of plasmids is their host range, i.e., the taxonomic breadth of bacterial species they can infect and from which they can be transferred (22, 23). Broad-host-range plasmids can spread across bacterial taxa, even spanning phyla, facilitating resistance gene transmission between commensals and pathogens alike (22–24). The hospital-based transmission of plasmids encoding the *blaOXA-48* gene between *Klebsiella* and *Escherichia* species has been documented, occurring both within and between patients (25). Furthermore, a study on neutropenic patients undergoing hematopoietic stem cell transplantation revealed plasmid transfer between bacteria from distinct phyla, predominantly Proteobacteria, Firmicutes, and Bacteroidetes (26).

The state of the lung microbiome is also a key factor in outcomes for critically ill patients. Notably, the presence of gut-associated microbial species in lungs correlates with poorer prognosis (27, 28). Severe illness and prolonged antibiotic exposure can lead to microbial translocation between body sites (28). The gut-lung axis plays a critical role in facilitating such transfers and may contribute to the dissemination of antimicrobial resistance. For instance, Wheatley et al. (29) demonstrated that lung colonization with *Pseudomonas aeruginosa* in an ICU patient originated from the gut. Meropenem treatment subsequently increased resistance in strains present in both sites (29, 30). Moreover, colonization of the gut with MDR *K. pneumoniae* was shown to exacerbate *P. aeruginosa* lung infections in mice by promoting gut dysbiosis (30). Conversely, bacterial lung infection in both mouse and human models disrupted the gut microbiota, reduced epithelial proliferation, and induced intestinal injury (19, 31, 32).

The fungal component of the microbiome—the mycobiome—comprises only a small fraction of total microbial biomass (0.1%–10%) but plays a crucial role in host-microbe dynamics (0.1%–10%) and is an important part of the microbial ecosystems of the human body (33, 34). The most abundant genera in the human gut mycobiome are *Candida*, *Saccharomyces*, and *Cladosporium* (35). Among these, *Candida* species, which represent a polyphyletic group, are consistently enriched in various disease states, especially within the gut lumen, and show an inverse relationship with prokaryotic diversity (35, 36).

Although common antibiotics target only prokaryotes, their use can indirectly promote fungal overgrowth, particularly of *Candida* spp., and lead to long-lasting alterations in the gut bacterial microbiota and mycobiota (37, 38). Recent studies have identified distinct shifts in the gut mycobiome of COVID-19 patients, independent of disease severity, with a notable overrepresentation of *Candida albicans* (39, 40). *C. albicans* can form synergistic biofilms with both pathogens and commensals, enhancing bacterial pathogenicity and providing protection against host defenses and antimicrobial agents (41). Similarly, *Nakaseomyces glabratus* (formerly *Candida glabrata*) can cause a spectrum of infections, from mucosal involvement to invasive candidiasis (IC), particularly in immunocompromised patients. IC originating from gut-resident *N. glabratus* is associated with high morbidity and mortality (42, 43).

To mitigate mortality from nosocomial infections, several protocols have been developed. One such strategy is selective digestive decontamination (SDD), which involves the administration of targeted antibiotics to the gut. While some studies have shown that SDD reduces secondary infections, its impact on overall mortality remains inconclusive (44, 45). Moreover, SDD can induce gut dysbiosis and encourage the emergence of resistant microbial strains, potentially worsening patient outcomes (46, 47).

Hi-C metagenomics is a powerful approach for studying complex microbial communities and has recently been applied to link bacterial hosts with mobile genetic elements in clinical settings (26, 48, 49). This method enables the identification of bacterial species putatively associated with plasmids within a microbiome by mapping Hi-C read pairs—representing spatially proximate DNA fragments—to an assembly of a conventional metagenome generated from the same biological sample. Because intra-cellular DNA ligation signals exceed inter-cellular ones, after statistical normalization of Hi-C junction counts, the chromosome-plasmid contacts from the same cell outcompete spurious intercellular associations. This approach is applicable to diverse metagenomes, as normalization suppresses background noise from unrelated cells, allowing us to explore the plasmid-host dynamics underlying the spread of antibiotic resistance.

In this study, we applied Hi-C metagenomics to stool samples from 11 critically ill COVID-19 patients to investigate their bacterial and fungal community composition. Using spatial contig interaction data, we constructed patient-specific plasmid transmission networks and mapped the distribution of AMR genes. We also compared metagenome-assembled genomes (MAGs) from stool samples to whole genomes of bacterial and fungal isolates from sputum, as well as analyzed bacterial plasmidomes to identify potential translocation events.

## MATERIALS AND METHODS

### Patients and gut microbiome sample collection

Stool and sputum (bronchoalveolar lavage [BAL]) samples were collected from 11 patients diagnosed with severe COVID-19 at Clinical Hospital #1, MEDSI. A subset of patients (*n* = 4) received SDD with the following protocol: oral cavity—application of 1 g paste containing 20 mg polymyxin B, 40 mg gentamicin, and 50 mg amphotericin; via nasogastric tube—140 mg polymyxin B, 300 mg gentamicin; and second- or third-generation cephalosporin administered for 3 days post-intubation. Clinical data are provided in Table S1.

### Hi-C metagenomic library preparation and sequencing

Whole-genome sequencing (WGS) and Hi-C metagenomic libraries were prepared from stool samples as previously described (49). Specifically, WGS metagenomic libraries were prepared using the NEBNext Ultra II FS DNA Library Prep Kit for Illumina per the manufacturer’s instructions. Total genomic DNA was isolated by cell lysis in 1× TE buffer (65°C, 14–16 h) with proteinase K (1 μg/μL) and 0.5% SDS, followed by phenol-chloroform extraction and ethanol precipitation (20 μg/mL glycogen). Residual RNA was removed with RNase A (25 μg, 37°C, 45 min), and salts with Agencourt AMPure XP beads. Input: 20–100 ng purified DNA.

For Hi-C libraries, briefly, the samples were resuspended in 0.9% NaCl, homogenized (FastPrep-24 with Lysing Matrix A), and fixed with 3% formaldehyde (22°C, 20 min), quenched with 125 mM glycine. Cells were lysed in an isotonic buffer (15 min on ice), and DNA was digested with HpaII (200 U total, 37°C, 16 + 2 h). The ends of DNA were filled in with biotinylated nucleotides by adding the pre-made mixture described in (50) and incubating for 75–90 min at 37°C. Ends were then ligated (T4 DNA ligase, 75 U, 16°C, 4 h). Cross-links were reverted (65°C o/n, proteinase K), and DNA was purified with phenol-chloroform, RNase A, and AMPure XP. The DNA was sheared to 100–1,000 bp (sonication), end-repaired, and A-tailed (Klenow exo-). The biotin pulldown was performed using Dynabeads MyOne Streptavidin C1 (Invitrogen), followed by ligation with TruSeq adapters (o/n, 22°C). The optimal number of PCR cycles (typically 10–12) was determined by test PCR on agarose gel; four preparative PCRs followed.

Sequencing was performed on the Illumina MiSeq platform using 150 bp paired-end reads.

### Bacterial and yeast strain isolation and sequencing

Bacterial and yeast isolates were obtained from sputum samples as follows: each sample was diluted in sterile saline, briefly centrifuged at 3,000 × *g*, and plated on Petri dishes containing selective nutrient media. Bacterial isolates were cultured in Luria-Bertani broth (Difco, Rockville, MD, USA) and on lactose 2,3,5-triphenyltetrazolium chloride (TTC) agar with Tergitol-7 (SRCAMB, Obolensk, Russia) at 37°C. *Candida* strains were grown on Sabouraud Maltose Agar (SRCAMB, Obolensk, Russia) under the same temperature conditions. Microorganisms were identified using a MALDI-TOF Biotyper instrument (Bruker, Karlsruhe, Germany), and isolates were stored in 15% glycerol at −80°C.

Genomic DNA was extracted using the CTAB protocol (51). WGS libraries were prepared with the MGIEasy Universal DNA Library Prep Set and sequenced on the MGIseq-2000 platform (MGI Tech Co., Ltd., Shenzhen, China). Short reads were assembled using Unicycler v0.4.7 (52). Genome annotation was performed using the NCBI Prokaryotic Genome Annotation Pipeline v5.3 (53).

### Preprocessing and prokaryotic profiling of metagenomes

Metagenomic data were processed following the workflow outlined in (49), with the following steps: WGS reads preprocessing: reads were merged, adapters removed, and sequences trimmed using SeqPrep v.1.3.2 (54) and Trimmomatic v.0.32 (55); taxonomic profiling (MetaPhlAn4 v.4.1.0 [56] and MiCoP [57]); assembly (metaSPAdes v.3.15.5 [58]); Hi-C reads preprocessing and filtering using BBMap’s v.37.62 bbduk (59); read alignment (BWA v.0.7.17 [60]) followed by processing in SAMtools v.1.3.1 (61); binning of contigs into MAGs: WGS-based (MetaBAT2 v.2.17 [62] and MaxBin2 v.2.2.7 [63]), as well as Hi-C-based (bin3c [64]); MAG taxonomic classification (GTDB-Tk v.2.4.0 [65]) and quality assessment (CheckM v.1.2.3 [66]); aggregation of prokaryotic MAGs (MetaWRAP v.1.3 [67] and DAS Tool v1.1.7 [68]). The proportion of valid Hi-C reads was assessed using qc3C v.0.5 (69).

### Plasmid-bacterium association: identification and statistical validation approaches

For the plasmid analysis, additional filtering was applied to the Hi-C reads. Initial assessment of noise-to-signal ratio for each sample was carried out using the following formula proposed in references 70, 71:


$$NSR\;=\frac{N_{inter\text{-}MAG}}{N_{intra\text{-}MAG}},$$

where *N*_inter-MAG_ is the number of Hi-C read pairs mapping to contigs from different MAGs, and *N*_intra-MAG_ is the number of Hi-C contacts originating from the same MAG. MetaWRAP-refined bins were selected for plasmid analyses based on high completeness and low contamination. One out of 11 samples exceeded the noise threshold defined at 0.2 (Table S2) and was excluded from subsequent plasmid analyses.

Then, following the approach described in reference 72, we considered “unreliable” and removed the read pairs that (i) mapped within 500 bp of a contig edge and (ii) mapped to the mobile genetic element sequences identified using ABRicate v1.0.1 (https://github.com/tseemann/abricate) and ISfinder database (73).

The remaining contacts were further filtered and normalized using HiCzin v.0.1.0 (74) with “-m nb” (negative binomial model) and “-t 0.15” (quantile threshold for spurious contacts detection) parameters, respectively. HiCzin normalizes metagenomic Hi-C data using zero-inflated negative binomial regression to model the intraspecies contacts, correct for biases (including coverage and restriction site frequency), and detect spurious interspecies links via enrichment scores and *P*-values.

Plasmid contigs were identified by Plasmer v0.1 (75). Antibiotic resistance genes in plasmid contigs were annotated using the Resistance Gene Identifier tool v.6.0.3 (76), which utilizes the Comprehensive Antibiotic Resistance Database (CARD) (77). AMR gene IDs reported are according to the CARD ontology. To associate plasmid contigs with potential bacterial hosts, we calculated average Hi-C contact frequencies between plasmid contigs and MAG contigs using bins with contamination lower than 10%. Pairs with <2 inter-contig Hi-C contacts were excluded. Contigs classified as viral by the viralVerify v.1.1 tool (78) were also removed from this analysis. All valid plasmid contig-MAG associations across the samples were merged. Taxonomic classification of MAGs was performed using GTDB-Tk.

Bacterium-plasmid links were validated using the MobReckon v.3.1.8 (79) plasmid incompatibility group annotations: taxonomy of each MAG linked to a plasmid was compared with the published data on the host range of the corresponding incompatibility group (23). The comparison confirmed that plasmid-MAG associations were consistent with known incompatibility group host scopes in each sample (Table S3).

A plasmid-sharing network was then constructed to represent the inter-genus interactions based on the shared plasmid contigs, with edge weights corresponding to the number of shared plasmid contigs. Visualization was conducted in R using the igraph package v.2.0.1.1 (80).

Plasmid transfer between lung and gut niches was verified using BLAST (81) alignment (≥90% identity, ≥90% coverage) of the pooled plasmids classified by Plasmer from both body sites.

### Bacterial comparative genomics and visualization

Comparative genome analysis was conducted using anvi’o v.8 (64), specifically the *anvi-pan-genome* function (parameters: --minbit 0.5 --mcl-inflation 10 --use-ncbi-blast), to compare MAGs, microbial isolates, and published reference genomes of *Klebsiella* strains. The bins selected for this analysis were obtained using MetaWRAP v.1.3. The phylogenetic tree was constructed using IQ-TREE v2.4.0 based on the Maximum Likelihood method (parameters: -m MFP + C20 -bb 1000 -alrt 1000 -T AUTO --wbtl -czb) (82). To compare two closely related *Klebsiella* strains, their genomes were aligned using MUMmer v.4 (83). Unaligned contigs were classified with viralVerify v.1.1 (78), and the genes they contained were annotated using Bakta v.1.10.3 (84). The longest contig was further aligned against the NCBI nucleotide database (85) to assess its likely viral origin. Average nucleotide identity (ANI) values were computed using *anvi-compute-genome-similarity* based on the pyANI package and visualized using the matplotlib (86) Python library.

Data visualization was performed using the R packages igraph v2.1.4, gplots v3.3.0, and ggplot2 v4.0.2. A tree for the MAG quality heatmap was constructed using the *anvi-gen-phylogenomic-tree* function, which is a wrapper script around the FastTree v2.11.1 (87) tool, which also uses Maximum Likelihood methods, based on MAGs representing a non-redundant species list. Gene sequences for tree construction were retrieved using the *anvi-get-sequences-for-hmm-hits* function with the Bacteria_71 HMM database. Tree visualization was performed using the phylobase v0.8.12 and phylogram R packages.

The *in silico* multi-locus sequence typing (MLST) was performed for the assembled genome sequences of *Acinetobacter baumannii* and *Klebsiella pneumoniae* isolates. Sequence types (STs) were determined using the command-line tool mlst v2.32.2 (https://github.com/tseemann/mlst). The analysis was executed with default tool parameters and used the integrated database schemes curated specifically for *A. baumannii* (Pasteur scheme) and *K. pneumoniae* (Pasteur scheme) as referenced from the PubMLST database (88).

### Fungal profiling

The fungal component of the microbiome was analyzed as previously described (50), with the following modifications. Taxonomic profiling was performed with Kraken2 v.2.1.4 (89) against the *core_nt* database (version June_2024) and additionally verified with BLAST v.2.16.0 against the *nt* database and KrakenUniq v.1.0.4 with the MicrobialDB database (90). WGS isolates were assembled with MEGAHIT v.1.2.9 (91). The *Candida* genomes were annotated using AUGUSTUS v.3.5.0 (92), with the built-in *S. cerevisiae* gene model applied to *N. glabratus* genomes and the *C. albicans* gene model applied to *C. albicans*. Drug resistance genes were identified based on the gene names following the approach outlined in reference 93. Additionally, protein sequences from fungal genomes were aligned against reference gene protein sequences from the Candida Genome Database (94) using BLAST. Dot plots were generated using D-GENIES v.1.5.0 (95) and minimap2 (96). The Hi-C read processing focused on *Candida* followed the workflow described in reference 50: chromosomal contact maps for MAGs were generated using Juicer v.1.6.0 (97); contigs were scaffolded using 3D-DNA v.201,008 (98), followed by manual refinement and reordering in JBAT v2.20.0 (https://github.com/aidenlab/Juicebox/wiki/Juicebox-Assembly-Tools). The quality of Hi-C-scaffolded assemblies was assessed using BUSCO v.5.0.0 (99), with the *saccharomyces-odb10* lineage data set. Phylogenetics was inferred by SCGs (single-copy genes) alignment with MUSCLE v.5 (100) and IQ-TREE (82) with 1,000 bootstrapping replicates and best-fit model automatic selection. The *in silico* MLST analysis for *C. albicans* and *N. glabratus* (Pasteur scheme used for both analyses) was conducted using the same tool (mlst v2.32.2) and approach as described in the “Bacterial comparative genomics and visualization” section above.

## RESULTS

### Gut dominance of bacterial opportunists and fungi in severe COVID-19

We analyzed stool samples from 11 patients with severe COVID-19, all of whom had been on mechanical ventilation for more than two weeks. Clinical metadata, including treatment regimens and pathogen screening results, are presented in Table S1. Samples from the first patient group (COV4–COV7) were collected between January and March 2021, while samples from the second group (COV1–COV3 and COV8–COV11) were collected from September to November 2021. For each patient, two types of sequencing libraries were generated: WGS (mean coverage 37 ± 19 million reads) and Hi-C (mean 44 ± 11 million reads; Table S2). The proportion of valid Hi-C read pairs was relatively low, ranging from 1% to 14%.

Preliminary prokaryotic community profiling, based on clade-specific marker genes, revealed pronounced dysbiosis characterized by a high relative abundance of opportunistic bacterial genera such as *Klebsiella*, *Escherichia*, *Enterococcus*, *Pseudomonas*, and *Streptococcus*. These genera accounted for up to 98% of total microbial abundance in individual samples—substantially higher than what is typically observed in healthy individuals (Fig. S1A).

Fungal profiling revealed that two samples (COV8 and COV9) exhibited abnormally high levels of yeasts not typically seen in healthy subjects. Specifically, several *Candida* species accounted for 28% and 39% of total read abundance, respectively, contrasting with low presence (mean < 0.01%) in the other samples (Fig. S1B; Table S5). These microbial features are characteristic of ICU patients and are associated with an elevated risk of sepsis and poorer clinical outcomes.

Four of the patients—those who underwent selective decontamination of the digestive tract (SDD; COV8–COV11)—manifested low diversity, with gut communities dominated by a single bacterial species (Fig. S1A): *GGB9063 SGB13982* species (*Scatosoma sp003150575* in the GTDB Release 226) in COV8 (84.4%), *Enterococcus faecalis* in COV9 (82.3%), and *Klebsiella pneumoniae* in COV10 and COV11 (96.1% and 51%, respectively). Similarly, in two non-SDD patients, we observed overrepresentation of a single taxon: *P. aeruginosa* in COV4 (56.1%) and *Parabacteroides distasonis* in COV7 (77.9%), reflecting extremely low community diversity.

**Fig 1:**
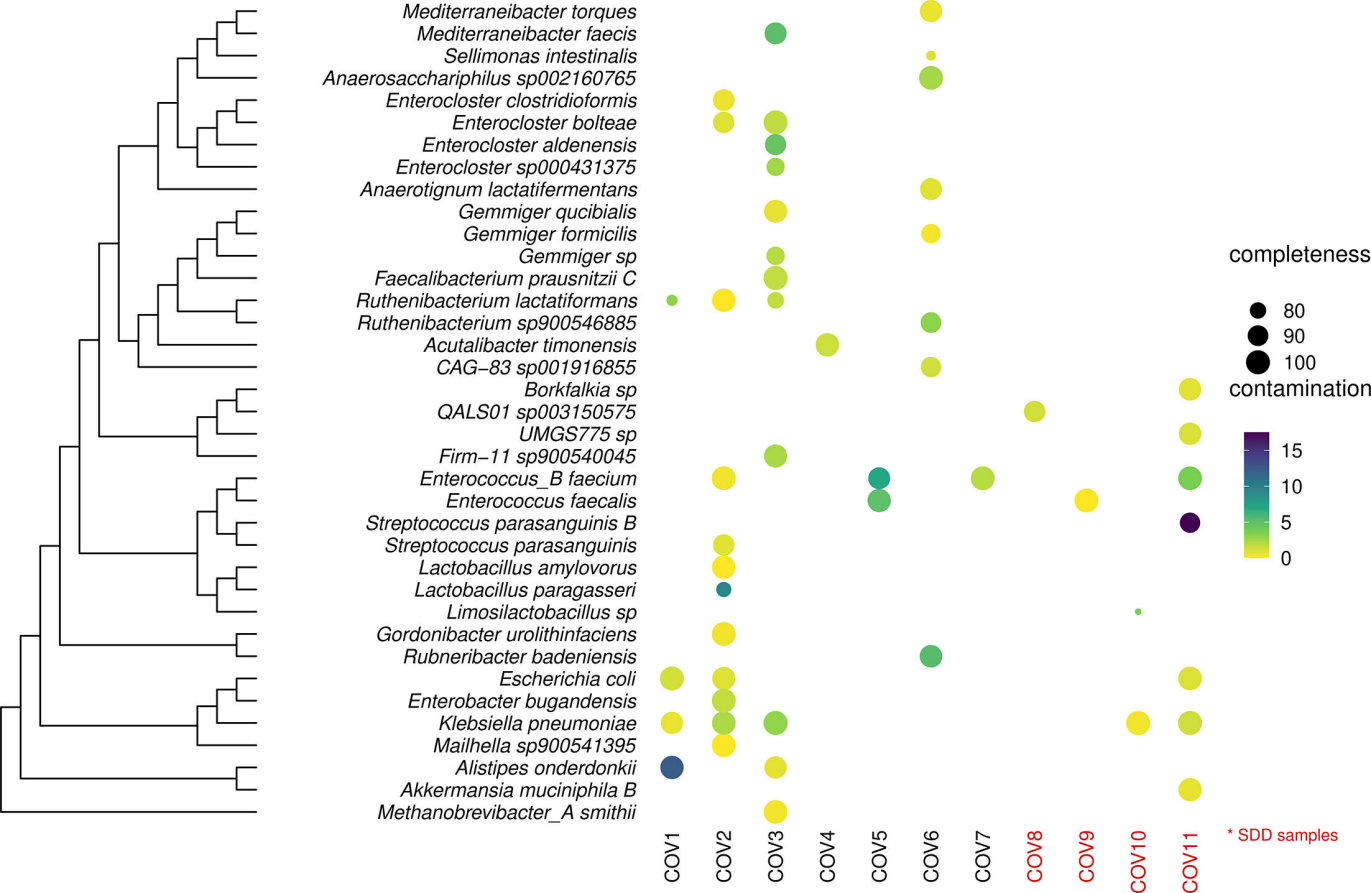
Reconstruction of prokaryotic MAGs from WGS and Hi-C data (aggregated). Shown are good-quality MAGs (completeness > 70% and contamination < 20%) obtained by combining outputs from two binning aggregators: MetaWRAP and DAS Tool. The MAGs are ordered by phylogeny (left), with circle size and color representing completeness and contamination, respectively.

To further characterize microbiome alterations associated with severe COVID-19, we reconstructed MAGs using three binning strategies: two based solely on WGS data (MetaBAT2 and MaxBin2), and one integrating WGS and Hi-C data (bin3c, referred to as Hi-C binning; see Materials and Methods). The number of reconstructed MAGs considerably varied between the metagenomes and was relatively low (mean eight MAGs per sample), with only four MAGs on average meeting medium-quality thresholds (completeness >70% and contamination <20%; Fig. 1; Fig. S2). In contrast to our earlier survey of ICU patients (49), the Hi-C bins in this study demonstrated lower contamination than their WGS-derived counterparts but showed no clear advantage in completeness. This likely reflects both the low diversity of the microbiomes and the relatively small proportion of valid Hi-C reads, which reduces the utility of Hi-C binning in reconstructing communities dominated by just a few taxa. Nonetheless, for two bacterial Hi-C MAGs—*E. coli* (sample COV1) and *Akkermansia muciniphila* (sample COV11)—coverage was sufficient to reconstruct chromosomal contact maps (Fig. S3). These maps displayed structural features previously reported for 3D bacterial genomes, including a main diagonal with evidence of chromatin macrodomains and smaller local chromosomal interaction domains. To enhance MAG quality, we applied DAS Tool and MetaWRAP aggregation methods to the combined set of WGS- and Hi-C-derived MAGs. This reduced contamination and modestly improved completeness (Fig. S2). Each tool exhibited a tendency to trade completeness for reduced contamination. The final refined MAGs yielded a species-level composition that was consistent with the results of marker gene-based profiling.

### Linking gut bacteria, plasmids, and drug resistance via Hi-C

We next examined bacterium-plasmid associations within the gut metagenomes of COVID-19 patients using Hi-C linkage data. Specifically, plasmid contigs were linked to chromosomal contigs from MetaWRAP-aggregated MAGs based on spatial proximity inferred from Hi-C read pair counts. Quality assessment based on the noise-to-signal ratio led to the exclusion of one sample, leaving 10 samples for this analysis (see Materials and Methods).

**Fig 2:**
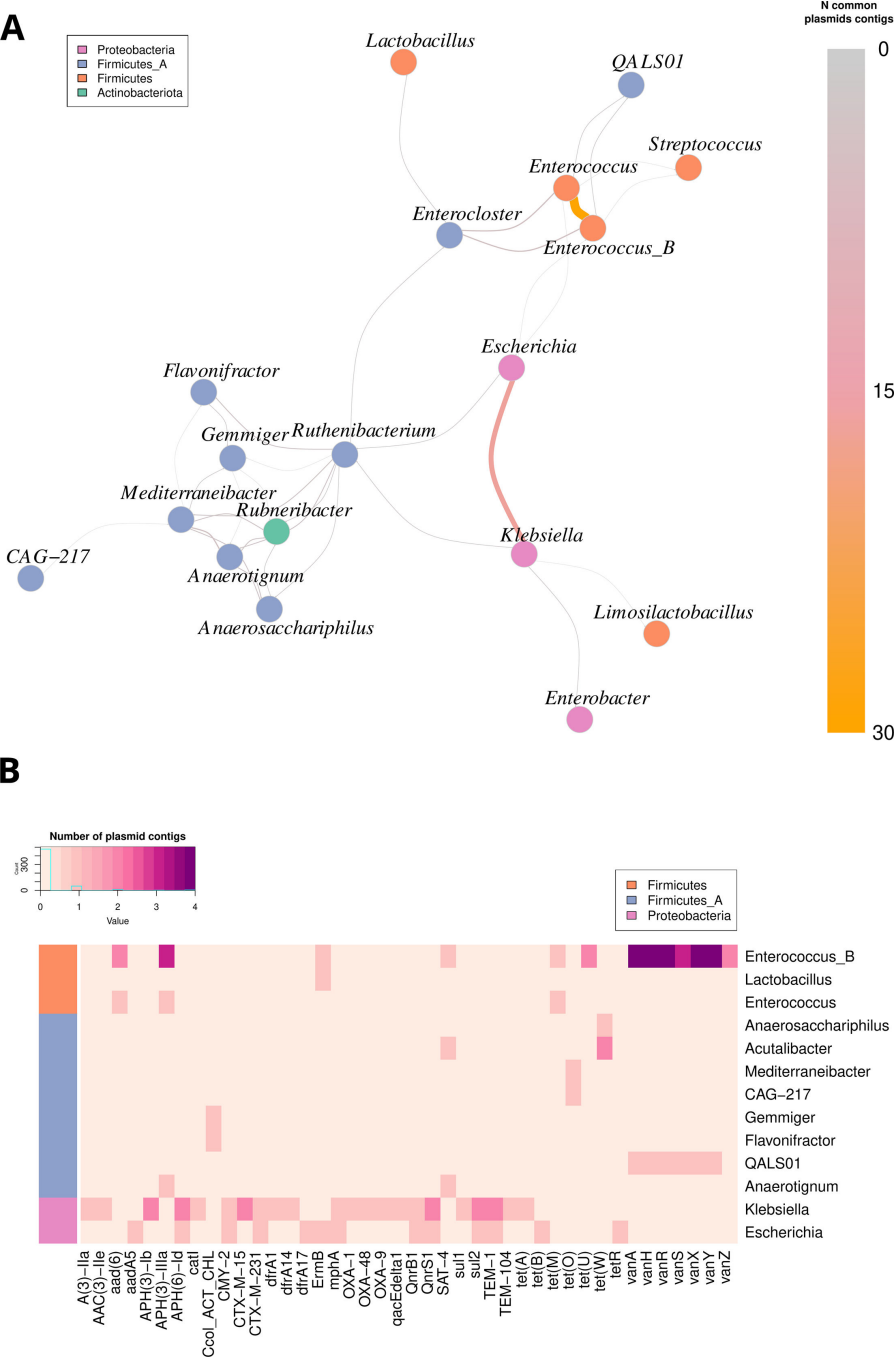
Gut plasmidome of critically ill COVID-19 patients and associated AMR profiles. (**A**) Inter-genus bacterial interactions via shared plasmids. (**B**) Antimicrobial resistance (AMR) genes identified on plasmid contigs.

Using validated plasmid-bacterium links, we constructed a network to visualize plasmid contig sharing across bacterial genera, thereby capturing potential plasmid transfer events (Fig. 2A). This network revealed plasmid-mediated connections among 18 bacterial genera spanning four phyla, exhibiting the expected intra-phylum clustering of bacterial hosts. The most extensive plasmid sharing occurred among genera within Proteobacteria, Firmicutes, and Firmicutes_A. Notably, there were shared plasmid contigs between known opportunist genera: *Enterococcus* and *Enterococcus_B* (*n* = 30 shared contigs), and *Klebsiella* and *Escherichia* (*n* = 17). We also observed interactions between opportunist and known commensal genera, such as *Klebsiella* with *Ruthenibacterium* and *Limosilactobacillus*.

To evaluate the potential for plasmid-mediated antibiotic resistance dissemination, we annotated AMR genes located on plasmid contigs (see Materials and Methods). Using Hi-C linkage information, we identified taxa potentially possessing the resistance profiles due to these plasmids (Fig. 2B). Overall, the plasmid contigs encoded resistance genes for 16 antibiotic classes, including clinically significant agents such as cephalosporins (*blaCTX-M* gene), carbapenems (*blaOXA-48*), and glycopeptides (*vanA* gene cluster). Eleven bacterial genera spanning three phyla were associated with AMR-bearing plasmids, with the majority of associations observed in *Klebsiella* and *Escherichia*.

We also detected evidence suggesting cross-genus and even cross-phyla dissemination of AMR genes. For example, the *van* gene cluster was found in Firmicutes_A genus *QALS01* (Borkfalkiaceae family; Fig. S4). Although vancomycin resistance gene dissemination has been well documented in *Enterococcus*, reports of its presence in other gram-positive genera remain scarce. However, recent findings suggest related *van* gene clusters may be distributed in non-enterococcal taxa, including Lachnospiraceae and Oscillospiraceae families (101). These observations underscore the importance of understanding resistance transmission pathways within the gut microbiota, particularly via horizontal gene transfer.

Due to the limited sample size, statistical assessment of correlations between AMR gene carriage and prescribed antibiotic therapy was not feasible. Nonetheless, several notable cases of plasmid-encoded resistance corresponding to administered drugs were identified (Table S6). Particularly, in patient COV1, an *E. coli*-associated plasmid harbored the *sul2* gene, which confers resistance to trimethoprim-sulfamethoxazole (biseptol). In COV2, a *K. pneumoniae*-linked plasmid encoded the *blaCTX-M-231* gene, conferring resistance to ceftazidime. In COV5 and COV7, both treated with vancomycin, *E. faecium*-associated plasmids carried the *vanA* gene cluster. Finally, in COV7, an *E. faecium* MAG was linked to a plasmid containing the *aad(6*) (synonym: ANT(6)) gene, conferring resistance to aminoglycosides, including gentamicin. Although none of the discussed plasmid contigs were associated with more than one MAG, one plasmid from sample COV1 was classified within the IncQ incompatibility group, known for its broad host range, suggesting the potential for dissemination across diverse bacterial taxa.

### Gut-lung comparison of drug-resistant bacteria and plasmids

In addition to stool samples, BAL samples were collected from seven patients (COV1–COV3 and COV8–COV11). Bacterial and yeast isolates were obtained from these specimens and subjected to whole-genome sequencing (see Materials and Methods), yielding on average two isolates per patient (Table S4). The availability of microbial isolate genomes from patient sputum samples allowed us to explore microbiome relationships between two clinically critical body sites in COVID-19—gut and lungs. Bacterial isolates included *K. pneumoniae*, *Proteus mirabilis*, *P. aeruginosa*, *Acinetobacter baumannii*, and other species associated with severe respiratory infections, particularly pneumonia (Table S4).

Given the clinical significance and pathogenic potential of *A. baumannii* and *K. pneumoniae*, sequence-based typing of their sputum isolates was performed. During the *in silico* MLST, all three isolates of *A. baumannii* and all two obtained isolates of *K. pneumoniae* were typed. For the former species, the isolates from COV1 and COV3 (strain internal IDs: B-9792 and B-9827, respectively) belonged to ST2 (ST-sequence type), while the isolate ID B-9828 from the COV11 sample was identified as ST78. For *K. pneumoniae*, the isolate ID B-9827 from the COV1 sample was assigned to ST147, and the isolate from COV11 (ID B-9817) was assigned to ST395.

**Fig 3:**
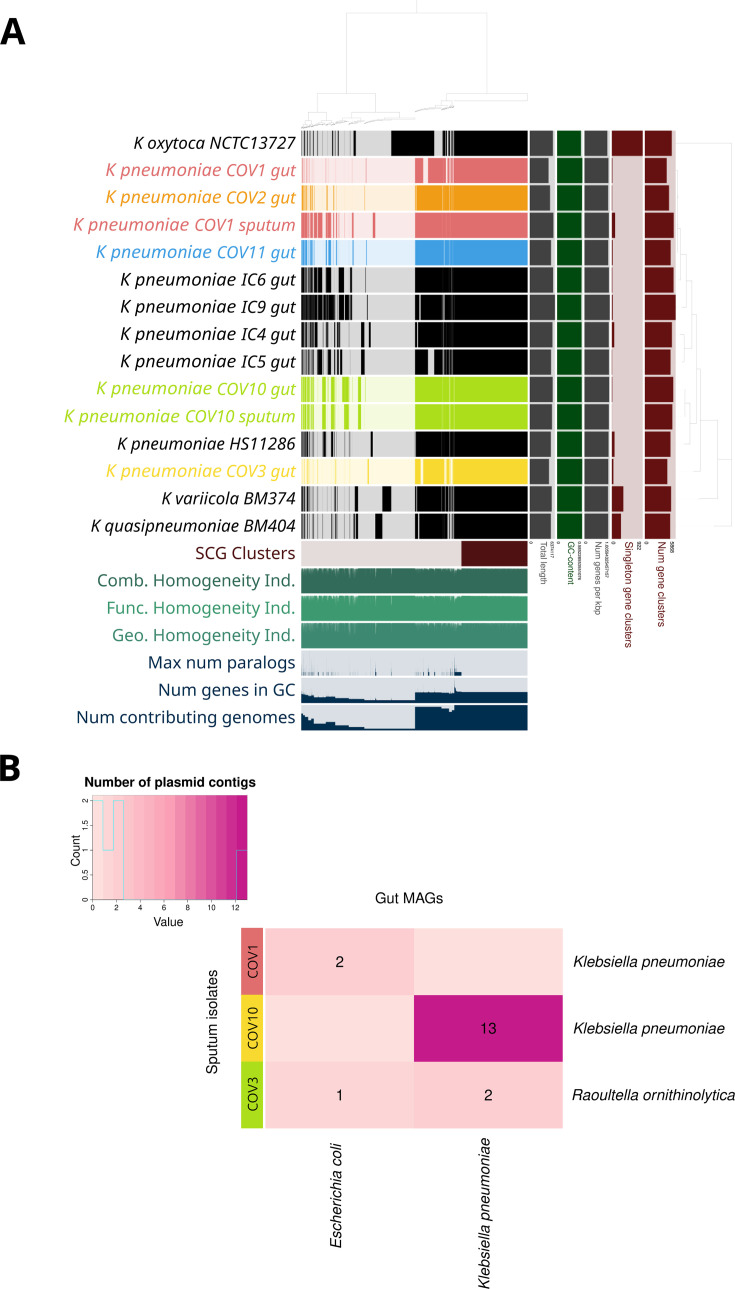
Comparison of bacterial genomes and plasmids from gut and lung samples. (**A**) Pangenome analysis of *Klebsiella* genomes obtained in this study alongside three reference strains (*K. pneumoniae* subsp. pneumoniae HS11286, *K. quasipneumoniae* BM404, and *K. variicola* BM374), and MAGs from Ivanova et al. (49). (**B**) Number of plasmid contigs shared between gut and lung microbiota within individual patients.

*Klebsiella pneumoniae* was the most prevalent species across both body sites. We yielded two of its genomes from lung isolates and four from stool MAGs. Pairwise comparisons among all these genomes (gut and lung) revealed high sequence identity (ANI > 99.2%; Table S7). For two patients (COV1 and COV10), both gut MAGs and lung isolates were available, allowing us to evaluate intra-subject strain-level correspondence between the two sites. To contextualize these findings, we augmented the genome set with gut-derived MAGs from long-stay ICU patients from our previous study (49) and reference genomes of four congeneric species (*Klebsiella oxytoca*, *Klebsiella pneumoniae* subsp. *pneumoniae*, *Klebsiella quasipneumoniae*, and *Klebsiella variicola*). Pangenomic analysis using anvi’o (102) revealed interesting clustering patterns (Fig. 3A). All new genomes fell within the *Klebsiella pneumoniae sensu stricto* clade but separated into subgroups.

Four genomes (gut and lung isolates from COV1, and gut genomes from COV2 and COV11) were highly identical and clustered with the long-stay ICU-derived *K. pneumoniae*. In contrast, gut and lung genomes from patient COV10 clustered closer to the reference strain HS11286. This patient’s microbiome was nearly monocolonized by *K. pneumoniae* (98% relative abundance; Fig. S1A), and the gut and lung genomes were nearly identical (ANI > 99.99%), differing by only a few single-copy gene clusters. Functional annotation of these genes revealed a prevalence of phage- and plasmid-associated genes, although without significant functional enrichment overall (Table S8). The longest differentiating contig (57 Kbp) aligned almost entirely (94% query coverage and 98.77% identity) with a *Caudoviricetes* bacteriophage genome (GenBank ID: BK046393.1; Table S9). These findings suggest translocation of the strain from the gut to the lung, or simultaneous colonization of the two sites from a shared source. The detection of highly similar *K. pneumoniae* strains across hospitals indicates circulation of a common clonal lineage or parallel colonization driven by similar clinical conditions.

In addition to bacterial genome comparisons, we investigated whether plasmids were shared between gut and lung bacteria. For each patient, plasmid contigs from gut and lung assemblies were aligned, and those with >90% alignment length were considered shared (see Materials and Methods). This allowed us to identify plasmids potentially transferred between bacterial taxa in different body sites (Fig. 3B). Plasmid overlap was observed in three of six patients. Notably, in patient COV10, *K. pneumoniae* shared 13 plasmid contigs between the gut and lung compartments, but in COV1, no plasmid contigs were shared. Interestingly, some plasmid contigs from gut *K. pneumoniae* were also aligned to *Raoultella ornithinolytica* isolates from the lungs, and *E. coli*-associated gut plasmids showed similarity to those found in both *K. pneumoniae* and *R. ornithinolytica* lung isolates for a few cases.

We then assessed the antibiotic resistance gene content of plasmids shared between the two body sites. In patients COV1 and COV10, AMR genes were detected on plasmids shared across gut and lung microbiota (Table S10). In COV1, plasmids shared between related species—gut *K. pneumoniae* and lung *E. coli*—carried the *blaCTX-M-231* and *qnrS-1* resistance genes, highlighting a potentially important route for AMR gene dissemination. Notably, *K. pneumoniae* was also detected in the bloodstream of patient COV1 (Table S1), suggesting systemic dissemination. In patient COV10, shared *K. pneumoniae* plasmids contained multiple AMR genes, including *tetA*, *qacE∆1*, *dfrA1*, *sul1*, *qnrS-1*, and *blaTEM-1*. These findings underscore the gut-lung axis as a potential channel for horizontal gene transfer and reveal possible pathways through which multidrug resistance may spread between host niches during critical illness.

### Co-existent *Сandida* yeast species across the lung and the gut

**Fig 4:**
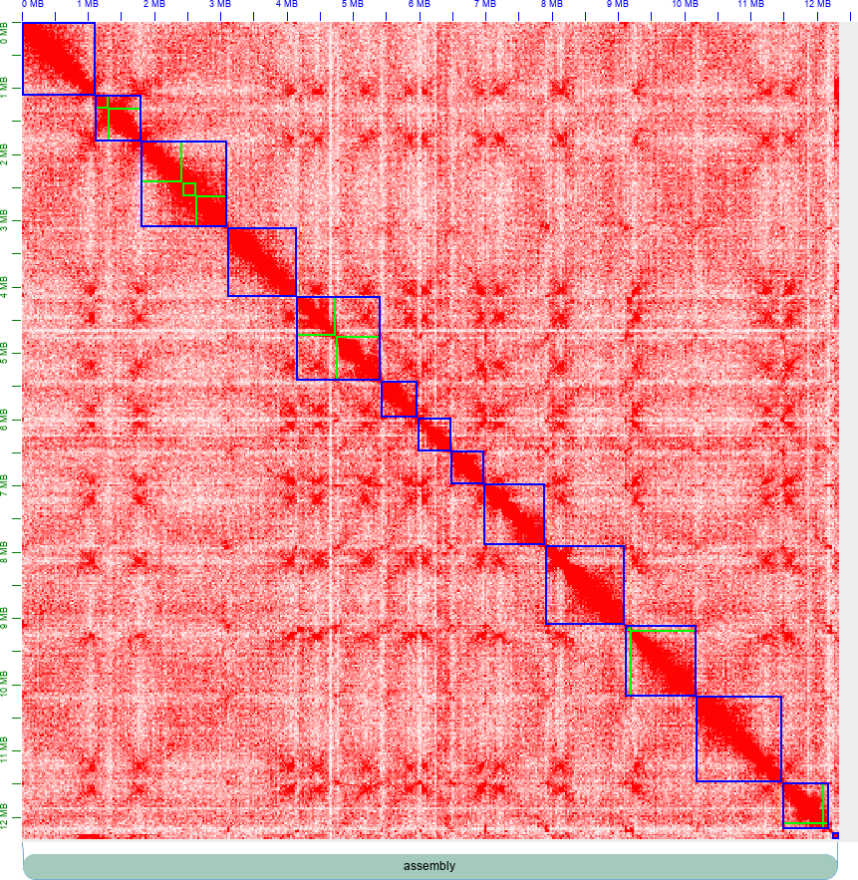
Chromosome contact map of *Nakaseomyces glabratus* Hi-C MAG from SDD2 gut metagenome. Contigs were scaffolded using 3D-DNA and manually reordered in JBAT. Green squares on the diagonal indicate large contigs; blue squares represent manually curated chromosome-scale scaffolds.

Initial taxonomic profiling of metagenomic reads revealed abnormally high levels of opportunistic *Candida* species in two samples—COV8 and COV9. To investigate this further, we applied our previously established Hi-C scaffolding approach for reconstructing fungal MAGs (103). In sample COV9, sequencing depth was sufficient to assemble a high-quality *N. glabratus* MAG with high completeness comparable to that of an isolate genome (BUSCO metric: 96.2%). The contact map of the *N. glabratus* MAG from the gut displayed 13 large quasi-chromosome scaffolds with distinct intra- and inter-chromosomal interaction patterns (Fig. 4), consistent with the known chromosome number for this species (104). Alignment with the reference strain *N. glabratus* CBS138 showed ANI of 97% and clear genomic synteny, with a few potential inversion rearrangements (Fig. S5), notably on chromosome L (GenBank accession number for *N. glabratus* CBS138 strain CP048129.1), which is in line with other reports (105), showing structural plasticity. For the COV8 sample, the gut-derived yeast genome assembly was of low quality due to insufficient coverage. However, from the same patient, we successfully cultured a lung *N. glabratus* isolate and generated a high-quality assembly (see Materials and Methods), which exhibited strong synteny (96.43%) with the reference genome *N. glabratus* CBS138 (Fig. S6). Mapping the gut metagenomic reads onto this sputum-derived genome revealed >99% alignment across the genome length, suggesting that the strains are nearly identical. Consequently, we constructed a contact map by combining the lung genome assembly with Hi-C data from the gut sample (see Materials and Methods). The resulting map resolved into 13 large chromosome-scale scaffolds (Fig. S7).

Additionally, we isolated *C. albicans* from the sputum samples of patients COV9 and COV11. These isolates were sequenced, assembled, and included in the comparative genomic analysis. Both assemblies were highly complete (96.3% and 96.4% BUSCO completeness for COV9 and COV11, respectively). In patient COV9, we detected concurrent colonization by both *C. albicans* and *N. glabratus*—a relatively uncommon finding that may reflect niche specialization within the host, influenced by the genomic features of each species that support colonization and survival. Such co-infections offer valuable insights into host–microbe interactions and fungal adaptive strategies.

**Fig 5:**
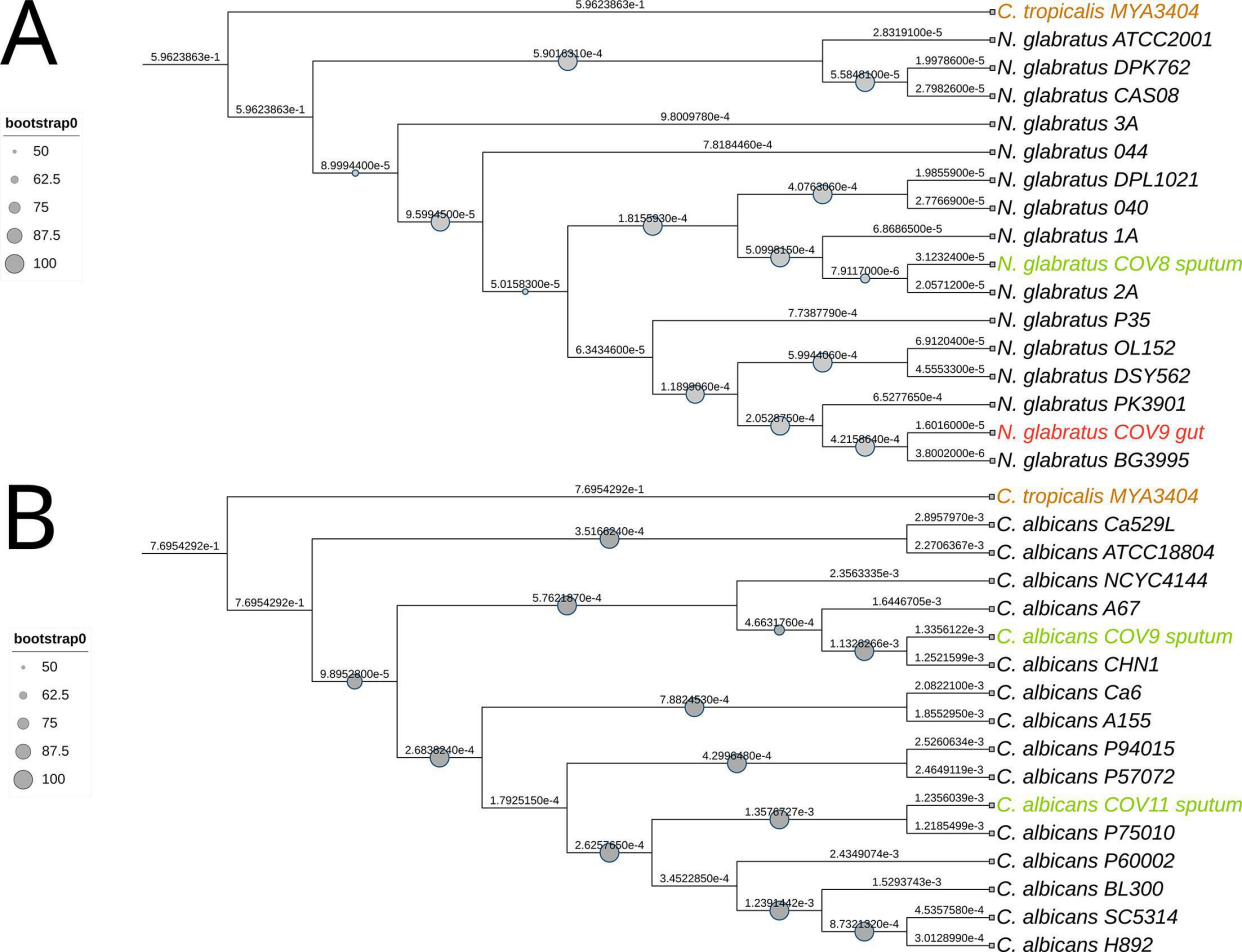
Phylogenetic trees of yeast genomes. Alignment of SCGs (single-copy genes) was performed in anvi’o and IQ-TREE and trees visualized in iTOL. (**A**) *N. glabratus* Hi-C MAG from the gut microbiome sample of patient COV9 and the isolated genome from the patient COV8 sputum among the published genomes. *C. tropicalis* MYA3404 was added as an outgroup. (**B**) *C. albicans* genomes isolated from sputum samples of COVID-19 patients COV9 and COV11 among the published genomes. *C. tropicalis* MYA3404 was added as an outgroup.

Fungal drug resistance is a critical determinant of poor outcomes, particularly in COVID-19 patients with fungal co-infections (106). All analyzed fungal genomes carried drug resistance genes (Table S11). Notably, the stool Hi-C MAG of *N. glabratus* from COV9 contained a gene (Candida Genome DB accession: CAGL0J01727g) known to confer resistance to multiple azole-class antifungals (93), reflecting adaptation to antifungal treatments and potentially increased risk to the patient. In addition to that, the lung isolate *N. glabratus* genome from the COV8 patient carried the fluorocytosine resistance FCY2 gene (107). To place these genomes in a broader context, we compared them against 14 publicly available *N. glabratus* genomes (Fig. 5A), selected from a wider set with a focus on clinical isolates (Table S12). A similar analysis was done for the sputum isolate genomes of *C. albicans* from patients COV9 and COV11 (with 14 published genomes of that species). The comparison showed similarity of our new genomes to particular clinical isolates (Fig. 5B). Finally, having conducted *in silico* MLST typing, similar to what was done for bacteria above, we were able to identify *N. glabratus* sputum isolate genome from the COV8 sample as ST6 and the *N. glabratus* gut MAG from the COV9 sample as ST46.

Although the genome assemblies of both *C. albicans* isolates (COV8 and COV11) exhibited high completeness, the *in silico* MLST analysis using the Pasteur scheme failed to assign a definitive ST. Notably, while the analysis for COV8 identified exact matches (100% identity) for all loci in the scheme, and analysis for COV11 detected a single nucleotide deletion within the ADP1_6 allele, resulting in a length of 442 bp compared to the canonical 443 bp sequence—a discrepancy that may reflect either a genuine genetic variant or an artifact of the assembly process. The specific combination of these allele numbers did not correspond to any ST profile currently cataloged in the PubMLST database. This outcome indicates that while the individual loci are conserved and well-represented in the reference database, the isolates may represent a novel allelic profile or a previously undescribed clonal lineage that falls outside the established classification framework.

Overall, our findings suggest that the gut and lung environments within the same patient can harbor genetically and functionally distinct fungal populations.

## DISCUSSION

In this study, we applied Hi-C metagenomics to dissect the microbiome structure, resistance gene mobility, and cross-body site microbial dynamics in critically ill COVID-19 patients. Our findings offer new perspectives on how intestinal and pulmonary microbiota may serve as interconnected reservoirs for multidrug-resistant organisms, and how genomic plasticity—particularly mediated by plasmids and niche-adaptive traits—might facilitate colonization and persistence across body sites.

A defining feature of the COVID-19 microbiomes was extreme dysbiosis, with some gut communities nearly monoclonal and dominated by known opportunists such as *Klebsiella pneumoniae*, *Enterococcus faecium,* or *Candida* spp. This pattern, particularly prevalent in patients undergoing SDD, raises questions about the microbiome resilience in the gut during intensive care. While SDD aims to suppress pathogen overgrowth, our observations from a limited number of SDD-treated patients suggest a possible association with increased susceptibility to colonization by antimicrobial-resistant strains. However, these findings should be interpreted with caution and verified in larger cohorts or through dedicated studies.

Hi-C metagenomics provides a dual benefit: enabling genome-resolved microbial profiling and allowing inference of *in situ* associations between mobile genetic elements and their bacterial hosts. This approach uncovered networks of resistance-carrying plasmids shared across genera and, notably, phyla. However, one cannot fully rule out the possibility of false positive plasmid-bacterium connections, stemming from ambiguous read mapping in chromosomal mobile element-rich regions, or excessive DNA ligation—particularly in samples with deviant biochemical properties; this could partially explain the high noise levels observed in one of our samples, which were subsequently filtered out. Overall, we adopted a conservative filtering strategy to favor minimizing false detections over maximizing true positive ones. An additional check using plasmid incompatibility groups showed that all of the inferred plasmid-host associations conformed to known host range constraints across most samples. At the same time, some potentially informative associations may have been lost due to this stringent processing. Evaluation of such cases could be improved in future studies by using defined “mock” communities of diverse plasmid-bearing species, spike-in control plasmids, or complementary experimental approaches such as those combining proximity ligation with super-resolution FISH imaging (26, 48, 72, 108). In total, the resulting network supports the notion that resistance dissemination within the gut is shaped by both plasmid compatibility and ecological opportunity, the latter often amplified in the ICU setting (10, 109).

Accurate plasmid annotation in short-read metagenomic data sets remains challenging due to repetitive elements causing assembly fragmentation and poor database representation of mosaic structures, while also suffering taxonomic bias, for example, with Enterobacteriaceae plasmids characterized better than those of *Enterococcus* (75, 110). Although specialized tools like Plasmer (75) attempt to mitigate these issues by integrating multiple features, the reconstruction of complete plasmids from short-read data remains suboptimal (110). Consequently, we opted to analyze high-confidence plasmid contigs rather than full plasmid assemblies. While long-read sequencing offers a potential solution for resolving these complex architectures, it remains cost-prohibitive for the high-coverage metagenomic depths required for this study.

Detailed genomic analysis suggested anatomical connectivity: plasmids and even near-identical bacterial strains appeared in both lung and gut samples of the same individuals. In some cases, these were accompanied by similar resistance gene profiles. These findings provide additional support for the hypothesis of a gut-lung microbial axis, where translocation of organisms or mobile elements may occur via aspiration and/or bloodstream dissemination facilitated by shared antibiotic selection pressures (111). In the context of COVID-19, such translocation is facilitated by intubation-related injuries common during high patient load. Notably, in one patient (COV1), a plasmid from gut *Klebsiella* was identified in lung *E. coli*, with no overlapping bacterial strain. Although the species are related and plasmid sharing between them has been reported before (25), such decoupling of plasmid and host supports the view that plasmids may serve as autonomous units of resistance spread across body compartments.

This aligns closely with the established mechanisms of the gut-lung axis, whereas gut dysbiosis compromises epithelial barrier integrity, enabling bacterial translocation through transcellular, paracellular, or dendritic cell-mediated pathways (28, 112). Recent studies reinforce this connection in animal models and clinical scenarios akin to ours. For instance, Wheatley et al. (29) tracked antibiotic-driven *P. aeruginosa* gut-to-lung translocation in an ICU patient, paralleling our identical strains/AMR profiles. Similarly, Le Guern et al. (30) showed that multidrug-resistant *K. pneumoniae* gut colonization worsens *P. aeruginosa* pneumonia in mice via translocation, and Coopersmith et al. (31) demonstrated pneumonia-induced gut dysbiosis feedback loops that perpetuate translocation risk, mirroring our *Klebsiella* plasmids in lung pathogens paired with severe dysbiosis. However, based on our data, it remains challenging to identify the primary origin of dysbiosis and highly pathogenic strains. We observe reciprocal dysbiosis and co-occurrence, suggesting translocation between the gut and lung, while studies incorporating multiple time points could elucidate the underlying causal relationships.

In hospital settings, where patients’ commensal strains acquire AMR plasmids amid microbiome depletion and immunity deficits, our findings emphasize tracking interspecies plasmid transfer. Time-resolved Hi-C metagenomics could elucidate these dynamics, integrating our plasmid-host decoupling with axis mechanisms and informing microbiome-targeted interventions to curb poor outcomes.

It is known that many hospital-acquired *Klebsiella* infections arise when a patient’s commensal strain acquires a plasmid enriched in AMR genes and, provided reduced protection from pathogens of the microbiome and immunity deficiency, increases the risk of poor outcome. In this context, tracking plasmid transfer across the species becomes even more important and should be further investigated through time-resolved Hi-C metagenomic analysis.

MLST is a key tool for defining pathogen population structure, identifying high-risk MDR and hypervirulent clones, while its *in silico* implementation enables direct typing from next-generation sequencing (NGS) data without extra assays. For *A. baumannii* sequence types, ST2 is the most prevalent lineage globally, driving carbapenem resistance (113) primarily via bla(OXA-23) (114). ST78 has also emerged as an epidemic clone (115). In *K. pneumoniae*, high-risk clones ST147 (carrying bla(OXA-48)/bla(NDM), capsular types K20/K64) (116) and ST395 (first outbreak in France, 2010) (117) spread resistance and hypervirulence. Thus, MLST surveillance is essential to track convergent clones posing a major public health threat.

Our analysis of *Candida* strains in gut and lung niches again raises the question of adaptation under antimicrobial therapy. More broadly, it exemplifies the role of fungal components in the clinical microbiome and the power of Hi-C metagenomics to uncover them with high resolution. It is important to note that obtained MAGs share similar high completeness and low contamination levels with genome isolates, proving the viability of using Hi-C to metagenomics data, thus being able to reconstruct genomes from metagenomics samples.

Interactions between fungal and bacterial microbiota may further influence colonization dynamics. Prior studies have shown that *Candida* spp. can promote or inhibit bacterial growth through biofilm formation and metabolic cross-talk (106), while gut anaerobes such as *Blautia* and *Roseburia* can inhibit *Candida* overgrowth via the production of short-chain fatty acids and other metabolites (118). Disruption of these interactions, especially under antibacterial pressure, may contribute to fungal expansion and resistance (119).

Earlier published MLST analysis of *N. glabratus* from Central Poland had identified ST6 as prevalent (13.3% of strains) (120), suggesting its clinical relevance. However, susceptibility testing showed no direct link between ST and azole MICs, and single-nucleotide polymorphism (SNP) analysis found no resistance-conferring SNPs (121, 122). Thus, ST6 resistance profile requires individual testing. In contrast, ST46 dominates elsewhere: a Kuwait study detected ST46 in 33/91 isolates across ≥4 hospitals (123), with no association to antifungal resistance but high prevalence and microevolution potential. Continued MLST surveillance is needed to track the spread of such clones in hospitals.

Our study is not free from limitations. The low number of patients, along with the challenges of preparing a consistently high-quality Hi-C library, constrain the depth of the results yielded during downstream data analysis. While our percentages of valid Hi-C reads (1%–14%) were generally in line with the published reports, they limited the ability to assemble complete MAGs (e.g., for the gut *N. glabratus* in the patient COV8) and plasmid linkage resolution. Nonetheless, even with these constraints, we assembled 61 bacterial genomes with a mean completeness of 86%, captured plausible plasmid-host associations, particularly comparable across the sites, and generated hypotheses with far-reaching implications. The inability to link specific treatments with resistance patterns—despite high-resolution genomic data—also underscores the complexity of microbiome-drug interactions. Similar difficulties were encountered in previous studies (26), though others have documented rapid selection for resistance following antibiotic exposure (25).

### Conclusions

Our work highlights the gut as both a reservoir and a hub for the evolution and transmission of antimicrobial resistance in critically ill patients. The combination of antibiotic pressure, disrupted microbial ecology, and genomic mobility creates a “perfect storm” for the spread of resistance across body sites and taxonomic boundaries. Hi-C metagenomics provides a powerful tool to study this ecosystem and may help guide more targeted interventions to prevent the expansion of resistance in the ICU and beyond.

Our study demonstrates the utility of Hi-C metagenomics for resolving complex microbial, plasmid, and resistance dynamics in critically ill COVID-19 patients. We revealed extensive dysbiosis, frequent dominance by opportunistic pathogens, and cross-body-site sharing of multidrug-resistant plasmids and strains. These findings support the concept of a gut-lung microbial axis and highlight the gut microbiome as a key reservoir for resistance gene dissemination. High-resolution, genome-resolved approaches such as Hi-C metagenomics are essential for understanding and ultimately mitigating the spread of antimicrobial resistance in clinical settings.

## Data Availability

The metagenomic sequencing data were deposited in the European Nucleotide Archive (ENA) under project accession PRJEB86834. The genome assemblies and raw data of the lung isolates were deposited in NCBI GenBank under BioProject PRJNA269675 (Table S4). Isolate internal strain IDs were provided along with the deposited genomes.

## SUPPLEMENTAL MATERIAL

Supplemental figures msphere.00139-26-s0001.pdf Figures S1 to S7.

Captions msphere.00139-26-s0002.docx Captions for supplemental tables.

Supplemental tables msphere.00139-26-s0003.xlsx Tables S1 to S12.

ASM does not own the copyrights to Supplemental Material that may be linked to, or accessed through, an article. The authors have granted ASM a non-exclusive, world-wide license to publish the Supplemental Material files. Please contact the corresponding author directly for reuse.
