# Supplementary material for: Gut Hi-C metagenomes of severe COVID-19 patients: bacteria and yeast involved in gut-lung axis
Source: mSphere. 2026 May 19;11(6):e00139-26. doi: 10.1128/msphere.00139-26 (PMC13317233; doi:10.1128/msphere.00139-26)
Supplement: Supplemental figures — Figures S1 to S7. [file msphere.00139-26-s0001.pdf]

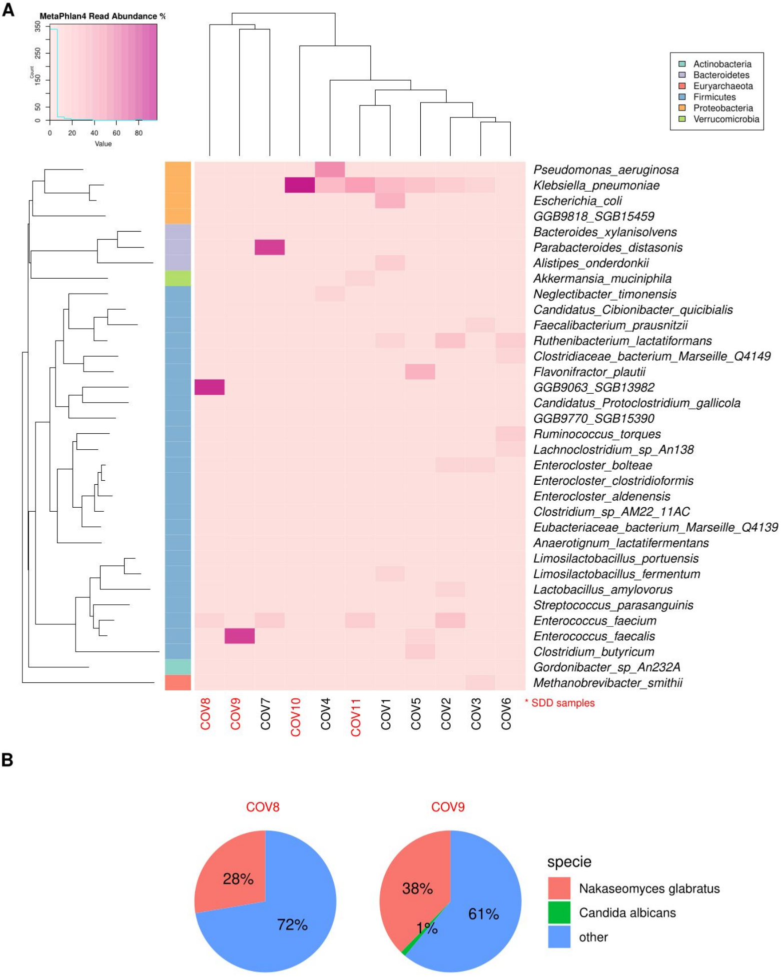

Suppl. Fig. 1. Taxonomic composition of gut metagenomes from COVID-19 patients. (A) Bacterial profiling using MetaPhlAn. (B) Fungal profiling using MiCoP (summary).

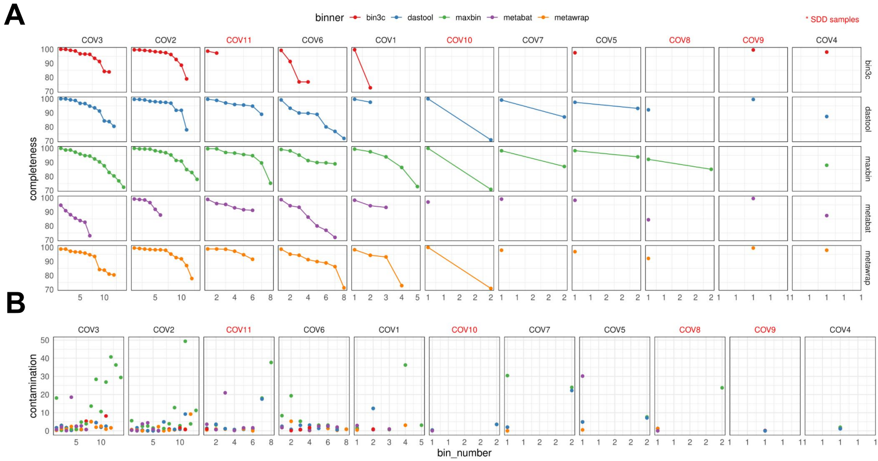

Suppl. Fig. 2. Comparison of MAG binning strategies applied to gut metagenomes. (A) Completeness and (B) contamination metrics are shown for three binners - bin3C (Hi-C-based), MaxBin2 and MetaBAT2 (WGS-based) - and two aggregation tools: DAS Tool and MetaWRAP.

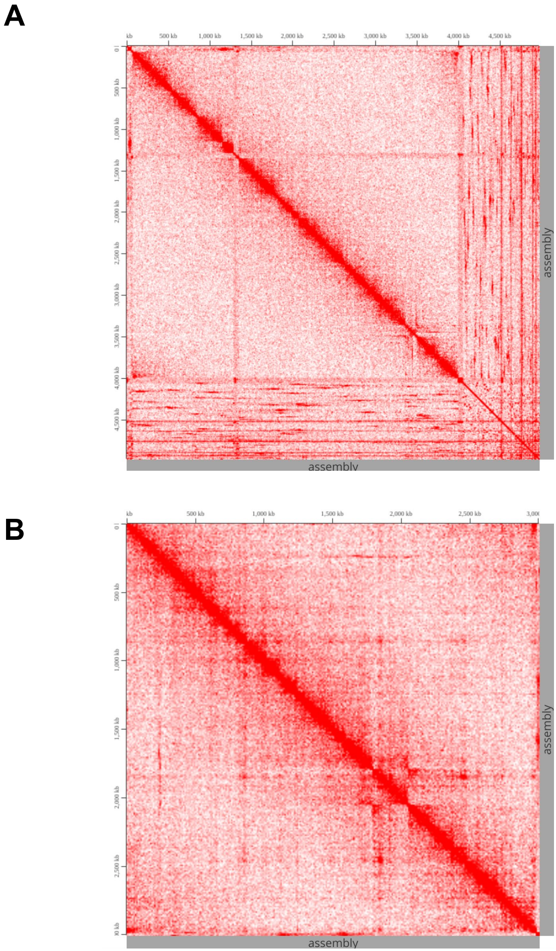

Suppl. Fig. 3. Chromosome contact maps of gut-derived bacterial species. (A) *Escherichia coli* MAG from patient COV1. (B) *Akkermansia muciniphila* MAG from COV11. Contigs were scaffolded using 3D-DNA. Visualized using Juicebox Web App (https://www.aidenlab.org/juicebox/).

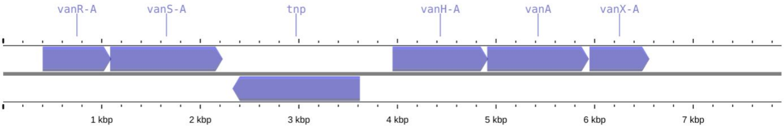

Suppl. Fig. 4. Localization of the *van* operon in plasmids linked to QALS01 genus (Borkfalkiaceae) MAG. Visualization made by Proksee Web App (https://proksee.ca/)

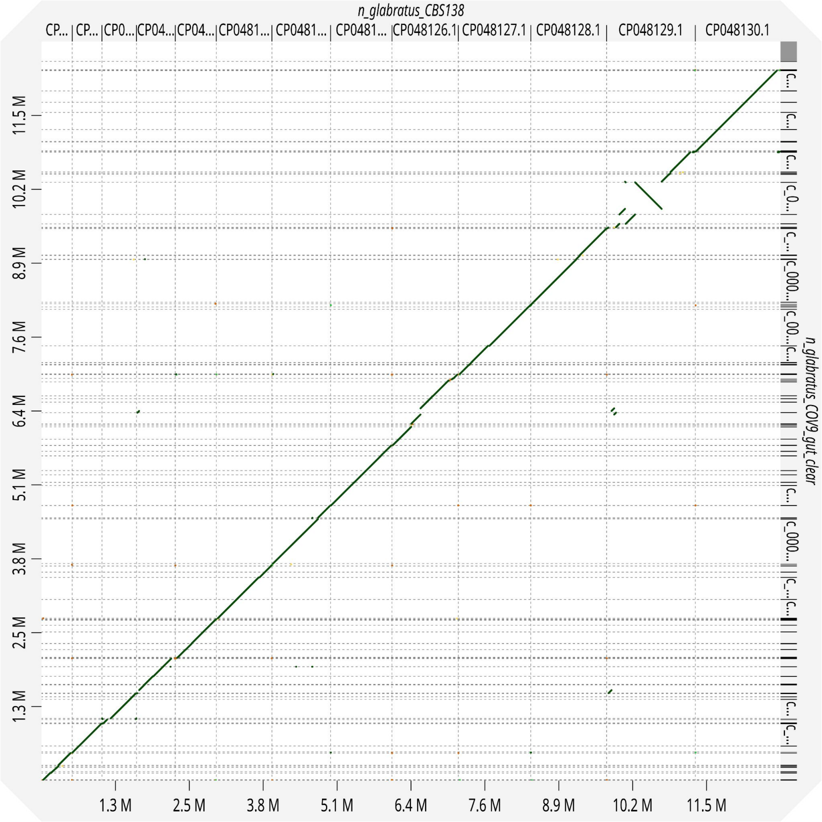

Suppl. Fig. 5. Dot plot comparing *N. glabratus* Hi-C MAG from COV9 gut sample to reference strain CBS138.

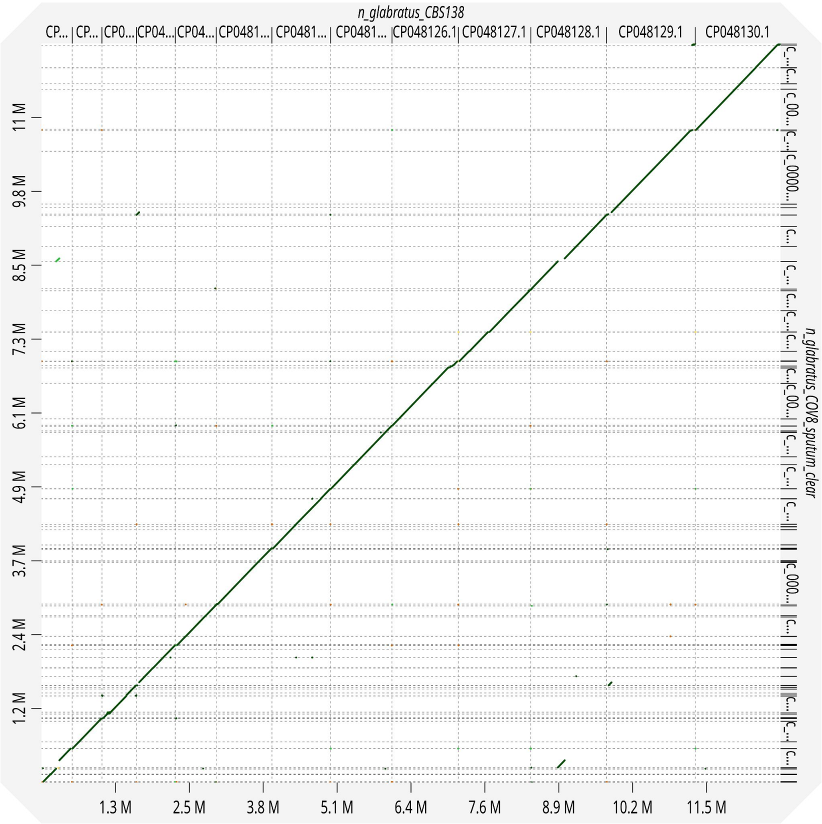

Suppl. Fig. 6. Dot plot comparing *N. glabratus* WGS assembly from COV8 sputum sample to reference strain CBS138.

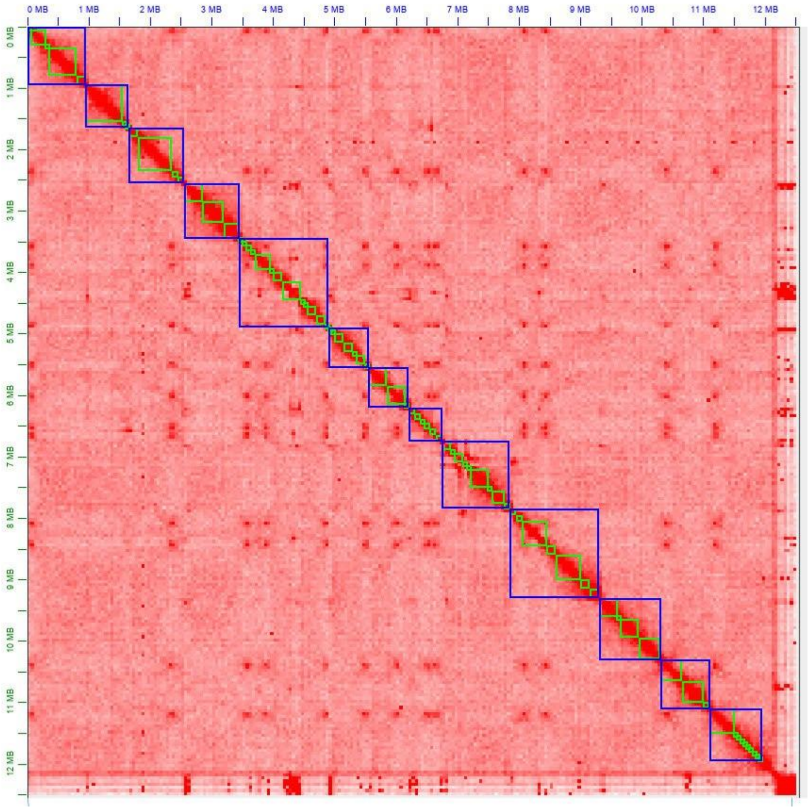

Suppl. Fig. 7. Chromosome contact map of *N. glabratus* WGS assembly from COV8 sample sputum isolate. Contigs were scaffolded using 3D-DNA and manually reordered in JBAT. Green squares on the diagonal indicate large contigs; blue squares represent manually curated chromosome-scale scaffolds.
