# Supplementary material for: Gut Hi-C metagenomes of severe COVID-19 patients: bacteria and yeast involved in gut-lung axis
Source: mSphere. 2026 May 19;11(6):e00139-26. doi: 10.1128/msphere.00139-26 (PMC13317233; doi:10.1128/msphere.00139-26)
Supplement: Captions — for supplemental tables. [file msphere.00139-26-s0002.docx]

**Suppl. Table 1.** Clinical data for the patients.

**Suppl. Table 2.** Metagenomic library statistics.

**Suppl. Table 3.** Predicted bacterial hosts and incompatibility group classifications of gut-associated plasmids.

**Suppl. Table 4.** Sputum isolates info.

**Suppl. Table 5.** Fungal community composition of the gut metagenomes, as determined by the MiCoP tool.

**Suppl. Table 6.** Instances where administered antibiotics matched the presence of corresponding resistance genes.

**Suppl. Table 7.** Average Nucleotide Identity (ANI) values among *Klebsiella* genomes from gut and lung samples.

**Suppl. Table 8.** Genes located on contigs that differ between the gut and lung Klebsiella genomes of patient COV10.

**Suppl. Table 9.** Contigs that differ between the gut and lung *Klebsiella* genomes of patient COV10.

**Suppl. Table 10.** AMR genes carried on common gut-lung plasmids.

**Suppl. Table 11.** Drug resistance genes identified in *Candida* genomes.

**Suppl. Table 12.** List of *Candida* genomes included in the pangenome analysis.
